# Supplementary material for: WordCommentsAnalyzer: A windows software tool for qualitative research
Source: F1000Res. 2018 Sep 4;7:536. Originally published 2018 May 3. [Version 2] doi: 10.12688/f1000research.14819.2 (PMC6137411; doi:10.12688/f1000research.14819.2)
Supplement: Supplementary file 3 [file f1000research-7-17614-s0002.tgz › b2a79496-4ada-47c4-9682-a99ce867836d.zip!/Eight Iranian Health Journals/The Journal of Toloo-e-Behdasht_13.docx]

© The Journal of Toloo-e-Behdasht. This work is licensed under CC BY 4.0 (https://creativecommons.org/licenses/by/4.0/).

Volume & Issue: دوره 15 - شماره 1

Title: جداسازی و تعیین اختصاصیت باکتریوفاژ بومی اشریشیاکلی از فاضلاب خام

http://tbj.ssu.ac.ir/article-1-2101-fa.html

مقدمه: باکتریوفاژها ویروس‌هایی هستند که بر سلول‌های پروکاریوت و به‌ویژه در تخریب باکتری‌ها اثرگذارند. مکانیسم اثر آن‌ها بسیار انتخابی است، به‌گونه‌ای که هر باکتریوفاژ تنها بر باکتری خاصی  اثر می‌گذارد. باکتریوفاژ‌ها به دلیل داشتن خواص ویژه،  می‌توانند جایگزین مناسبی برای آنتی‌بیوتیک‌ها در درمان بیماری‌های عفونی باشند. هدف این تحقیق جداسازی باکتریوفاژ مخصوص باکتری اشریشیاکلی از فاضلاب است. روش بررسی: هشت نمونه از فاضلاب که هرکدام شامل حدود 50 میلی‌لیتر فاضلاب خام بود و بافاصله زمانی 10 دقیقه از تصفیه‌خانه زرگنده واقع در تهران جمع‌آوری شد. سپس با محیط کشت Brain-Heart Infusion Medium) (BHI: به‌عنوان محیط کشت مایع جهت رشد میکروارگانیسم‌ها مخلوط شد. مراحل دیگر کار شامل خالص‌سازی ، تلقیح باکتری و تعیین میزبان نیز برای جداسازی باکتریوفاژ انجام شدند. جهت انتخابی بودن تأثیر باکتریوفاژ بر روی میکروارگانیسم‌ها‌یی مانند:E.coli (ATCC25922)، Enterococcus faecalis (ATCC19433)، Staphylococcus aureus (ATCC2392) وYersinia enterocolitica (ATCC9610) بررسی شد. یافته‌ها: از هر هشت نمونه باکتریوفاژ منتخب اشریشیاکلی جدا شد.آزمایش تعیین میزبان نشان داد که این فاژها، به‌خوبی قادر به لیز کردن و از بین بردن باکتری اشریشیاکلی هستند، اما روی سایر باکتری‌های مورداستفاده، هیچ تأثیری ندارند. نتیجه‌گیری: نتایج به‌دست‌آمده نشان داد که باکتریوفاژها به‌صورت اختصاصی عمل می‌کنند. با توجه به اینکه میزان مقاومت به آنتی‌بیوتیک‌ها در حال افزایش است، باکتریوفاژها می‌توانند جایگزین مناسبی برای کاربرد آنتی‌بیوتیک‌ها در درمان عفونت‌های باکتریایی باشند.

Title: کاربرد روشهای WilliamFine , PHA در مدیریت ریسک مخاطرات زیست محیطی و برآورد هزینه های کنترلی در مجتمع فولاد کویر

http://tbj.ssu.ac.ir/article-1-2102-fa.html

چکیده مقدمه: در صورت عدم کنترل صحیح مخاطرات و ریسک های زیست محیطی ناشی از صنایع ، می تواند خسارات جبران ناپذیری به محیط زیست وارد گردد . از این رو ، این مطالعه با هدف شناسایی و ارزیابی ریسک های زیست محیطی ( نظیر: انتشار آلاینده ها به هوا،  تخلیه آلاینده ها به آب ، تخلیه آلاینده ها به خاک ، مصرف مواد خام، مصرف انرژی و...) و برآورد هزینه های کنترلی به روش ویلیام فاین (william fine) و (PHA) در مجتمع فولاد کویر صورت گرفته است. روش بررسی: پس از دسته بندی مخاطرات زیست محیطی در لیست مقدماتی خطر((PHL: Preliminary Hazard List براساس واحدکاری، فعالیت های انسانی و عملکرد ماشین آلات  موجود در سالن تولید مجتمع فولاد کویر به صورت مطالعات میدانی شناسایی وارزیابی ریسک به روش ویلیام فاین جهت RAC1 انجام شد. راهکار اصلاحی برای ریسکهای بحرانی ارائه وRAC2  محاسبه گردید. در این تحقیق از نرم افزار SPSS  نسخه19 جهت محاسبات آماری استفاده شد. یافته ها : براساس یافته های این تحقیق، تعداد 356خطر زیست محیطی، در قالب 9 گروه خطر شناسایی شد. قبل از مداخلات، تعداد ریسک های قابل قبول89، پایین ترین حد قابل قبول ALARP )) 204و ریسکهای غیرقابل قبول (بحرانی) 63 بود. پیش بینی می شود پس از اجرای اقدامات کنترلی تعداد ریسک های قابل قبول به 183 و پایین ترین حد قابل قبول به 173 تغییر یابد. نتیجه گیری : نتایج این مطالعه نشان می دهد که شناسایی جامع و ارزیابی مخاطرات زیست محیطی در صنعت نورد، ضمن نمایش معایب و نواقص فنی مخرب محیط زیست و خطاهای پرسنل نسبت به این مقوله ، نیاز به بازرسی های زیست محیطی، آگاه سازی و حساس سازی و تغییر نگرش پرسنل شاغل در این صنعت را نسبت به حفظ و ارتقاء سلامت محیط زیست ، ضروری می سازد. این مقاله حاصل پایان نامه کارشناسی ارشد بهداشت محیط، دانشگاه علوم پزشکی شهید صدوقی یزد است.

Title: بررسی مشکلات خواب در دانش‌آموزان مدارس ابتدایی شهر کاشان در سال1392

http://tbj.ssu.ac.ir/article-1-2103-fa.html

مقدمه: مشکلات خواب در کودکان سنین مدرسه شایع می‌باشد که علیرغم شیوع بالا، مشکلات خواب اغلب نادیده گرفته می‌شود. هدف از این مطالعه تعیین فراوانی مشکلات خواب در دانش‌آموزان مقطع ابتدایی شهر کاشان می‌باشد. روش بررسی: این تحقیق یک مطالعه توصیفی- مقطعی است که 300 نفر از کودکان سنین 7-12 سال مدارس ابتدایی شهر کاشان موردبررسی قرار گرفتند. نمونه‌گیری به روش تصادفی از نوع خوشه‌ای و ابزار گردآوری داده‌‌ها سؤالات مربوط به سن و جنس کودک به همراه پرسشنامه BEARS که توسط مادران کودکان موردمطالعه تکمیل شد. جهت تجزیه تحلیل داده‌ها از روش آمار توصیفی (میانگین و انحراف معیار) و آزمون T-Test نمونه‌های مستقل برای مقایسه میانگین طول مدت خواب و آزمون من ویتنی یو تست برای بررسی اختلاف بین گروه‌های سنی و جنسی استفاده شد. یافته‌ها: درمجموع 300 نفر در این مطالعه شرکت کردند که 3/44 % پسر و 7/55% دختر بودند. شایع‌ترین مشکلات خواب سخت بیدار شدن از خواب و کابوس شبانه بود. فراوانی نسبی بیدار شدن در طی شب در پسرها بیشتر از دختران بود (05/0P<) . نتیجه‌گیری: فراوانی بالای مشکلات خواب در گروه موردمطالعه می‌تواند نشانگر اهمیت توجه به مشکلات خواب در دانش‌آموزان و اطلاع‌رسانی در خصوص الگوهای بهداشتی خواب به‌منظور ارتقا سطح آگاهی والدین از طریق مدارس و جلسات اولیا و مربیان باشد.

Title: بررسی حذف رنگ متیلن‌بلو از محلول‌های آبی با استفاده ازپودر پوست بادام زمینی

http://tbj.ssu.ac.ir/article-1-2105-fa.html

چکیده مقدمه: صنایع نساجی یکی از بزرگ‌ترین تولیدکنندگان ترکیبات آلی و رنگی می‌باشند. متیلن‌بلو رایج‌ترین ماده رنگی جهت رنگ‌آمیزی است. در مطالعه حاضر، پودر پوست بادام‌زمینی به‌عنوان یک جاذب ارزان‌قیمت جهت حذف رنگ متیلن‌بلو از محلول‌های آبی مورداستفاده قرارگرفته است. روش بررسی: در این مطالعه تجربی، در مقیاس آزمایشگاهی اثر پارامترهای بهره‌برداری ازجمله  pH(12-2)، زمان تماس (210-5 دقیقه)، مقدار جاذب (1-1/0 گرم بر لیتر) و غلظت اولیه رنگ (120-10میلی‌گرم بر لیتر) بر حذف رنگ از فاضلاب سنتتیک موردبررسی قرار گرفت. همچنین رفتار جذب رنگ‌ توسط ایزوترم‌های فروندلیچ و لانگمویر ارزیابی شد. یافته‌ها: بالاترین راندمان حذف رنگ متیلن بلو از محلول در pH بهینه11، زمان تماس75 دقیقه و دوز جاذب 8 گرم برلیتر و غلظت اولیه 10 میلی‌گرم بر لیتر حاصل شد. وقتی‌که غلظت از 10 به 100  میلی‌گرم بر لیتر می‌رسد راندمان حذف از 5/99 به 46/96 درصد کاهش می‌یابد. نتایج بررسی ایزوترم‌های جذب نشان داد که فرآیند جذب متیلن‌بلو با استفاده از پودر پوست بادام‌زمینی از مدل لانگمویر (R2=0.894) پیروی می‌کند. نتیجه‌گیری: بر اساس نتایج این مطالعه، پودر پوست بادام‌زمینی از توانایی مطلوبی در حذف رنگ متیلن‌بلو از محلول‌های آبی و فاضلاب‌های صنعت نساجی برخوردار است.

Title: بررسی غلظت فلزات سنگین کروم و آرسنیک در رودخانه‌های حوضه سدهای بافت و رابر

http://tbj.ssu.ac.ir/article-1-2104-fa.html

مقدمه: فلزات سنگین از عناصر سمی و پایدار در محیط هستند که با ورود به منابع آبی نهایتاً وارد چرخه زیستی جانداران شده و اثرات نامطلوبی ایجاد می‌نمایند. پژوهش حاضر جهت بررسی تغییرات غلظت فلزات سنگین کروم و آرسنیک در رودخانه‌های حوضه سد بافت و سد رابر که بعضاً به مصرف شرب رسیده و یا به این منظور پیش‌بینی‌شده‌اند، می‌پردازد. روش: مطالعه حاضر از نوع توصیفی است که از تابستان 1392 لغایت بهار سال 1393 انجام گرفت. طی بازدید میدانی 4 ایستگاه نمونه‌برداری از رودخانه‌های حوضه سدهای بافت و رابر انتخاب شد. تعداد 1 نمونه مرکب از رودخانه‌های مذکور و سدهای بافت و رابر در نیمه هرماه برداشت شد. غلظت فلزات سنگین کروم و آرسنیک در رودخانه‌های (بافت، سلطانی) حوضه سد بافت، و رودخانه‌های (رابر، سرمشک، سید مرتضی، رود در) حوضه سد رابر، به مدت 12 ماه اندازه‌گیری شد. غلظت کروم و آرسنیک با جذب اتمی کوره گرافیتی سنجش شد. داده‌ها با نرم‌افزار spss تجزیه‌وتحلیل شد. یافته‌ها: میانگین غلظت کروم در رودخانه‌های حوضه سد بافت به ترتیب 01/5 و 19/5 ppb و در رودخانه‌های حوضه سد رابر به ترتیب 44/5، 5/5، 42/5 و 45/5 ppb به دست آمد. میانگین غلظت آرسنیک در رودخانه­های حوضه سد بافت به ترتیب 52/16 و 71/11 ppb و در رودخانه‌های حوضه سد رابر به ترتیب 28/12، 6/13، 07/13 و 78/8 ppb حاصل شد. میانگین غلظت کروم در سدهای بافت و رابر به ترتیب 02/5 و 38/5 ppb و آرسنیک به ترتیب 53/23، 12/9 ppb به دست آمد. نتیجه‌گیری: بر اساس نتایج به‌دست‌آمده غلظت فلز کروم در ایستگاه‌های موردمطالعه از رهنمود سازمان جهانی بهداشت، حد مجاز استاندارد سازمان حفاظت محیط‌زیست آمریکا و حد مجاز موسسه استاندارد تحقیقات صنعتی ایران کمتر بود. غلظت فلز آرسنیک از رهنمود سازمان جهانی بهداشت، حد مجاز استاندارد سازمان حفاظت محیط‌زیست آمریکا و از حد مجاز موسسه استاندارد تحقیقات صنعتی ایران بیشتر بود. این تفاوت ازنظر آماری معنی‌دار بود (0001/0p<). با توجه به اهمیت این موضوع ضرورت دارد که این مسئله رسیدگی و در ارتباط با تقلیل آن برنامه‌ریزی‌های لازم به عمل آید.

Title: مقایسه هوش معنوی و سبک‌های اسنادی در زنان معتاد و غیرمعتاد

http://tbj.ssu.ac.ir/article-1-2106-fa.html

چکیده مقدمه: هوش معنوی به درک رابطه با سرچشمه هستی و معنا بخشی به زندگی گفته می‌شود؛ یعنی آنچه هدف غایی ما از زیستن است را تشکیل می‌دهد. این پژوهش باهدف مقایسه هوش معنوی و سبک‌های اسنادی زنان معتاد و غیر معتاد شهر یزد انجام‌شده است. روش بررسی: این پژوهش این پژوهش از نوع همبستگی بود. جامعه موردمطالعه این پژوهش شامل کلیه زنان شهر یزد در سال 1392 بود؛ که نمونه‌ای به حجم 300 نفر به روش نمونه‌گیری خوشه‌ای تصادفی چندمرحله‌ای از مناطق سه‌گانه شهر یزد انتخاب شد؛ و از جامعه زنان معتاد مراجعه‌کننده به مراکز ترک اعتیاد در شهر به روش نمونه‌گیری خوشه‌ای چندمرحله‌ای تصادفی نمونه‌ای با حجم 108 نفر انتخاب شد. در این پژوهش از پرسشنامه هوش معنوی 29 سؤالی عبدالله زاده وهمکاران و از پرسشنامه سبک اسنادی پترسون و سلیگمن(ASQ) استفاده شد و داده‌ها با روش‌های آماری‌ تی مستقل و همبستگی پیرسون تحلیل شد. یافته‌ها: نتایج پژوهش بیانگر تفاوت معنادار میان هوش معنوی زنان معتاد و غیر معتاد بود (یک دامنه، 05/0>P، 406=df، 22/3=t)، همچنین زنان معتاد به‌طور معناداری از سبک اسنادی منفی در برابر شکست‌ها استفاده می‌کردند (یک دامنه، 001/0>P، 406=df، 90/5=t) و بین هوش معنوی و سبک‌های اسنادی همبستگی مثبت وجود دارد (582/0=rs، 306=N، 001/0>P، دو دامنه). نتیجه‌گیری: بر اساس یافته‌های پژوهش، بدون درک معنای زندگی، زیستن ارزش چندانی برای انسان نخواهد داشت. چراکه رنج زندگی بدون معنویت، رنج معنادار نخواهد بود؛ و در مواجهه با چالش‌های زندگی انسان را به کناره‌گیری و درماندگی خواهد رساند. بر همین اساس یافتن معنای زندگی و داشتن سبک‌های اسنادی مثبت از عوامل اثرگذار بر گرایش افراد به مواد مخدر است.

Title: بررسی کارایی نانو ذرات مغناطیسی پوشش داده شده با پلیمر کاتیونی کیتوزان در حذف کدورت از محلول‌های آبی

http://tbj.ssu.ac.ir/article-1-2107-fa.html

چکیده مقدمه: کدروت آب به دلیل وجود ذرات، ترکیبات آلی و میکروارگانیسم‌ها بوجود می‌آید. مطالعه حاضر به منظور بررسی کارایی نانو ذرات مغناطیسی پوشش داده شده با پلیمر کاتیونی کیتوزان در حذف کدورت از محلول‌های آبی طرح‌ریزی گردید. روش بررسی: این مطالعه از نوع مداخله‌ای آزمایشگاهی می‌باشد. در این مطالعه از تکنیک ترسیب شیمیایی همزمان جهت سنتز نانو‌ذرات مغناطیسی و پوشش آن با پلیمر کیتوزان استفاده شد و اثرات دوز نانو‌ذرات مغناطیسی(4 و 3، 2، 1 گرم در لیتر)، pH (در محدوده 9 و7،6 ،4)، زمان ته‌نشینی(60، 50، 40، 30، 20، 10 و 5 دقیقه)، قدرت میدان مغناطیسی خارجی(در محدوده 7/0 و 5/0، 25/0، و صفر تسلا) و مقادیر کدورت‌های اولیه مختلف(NTU 1000و 750، 500، 250) بررسی گردید. یافته‌ها: نتایج نشان داد که افزایش دوز نانو‌ذرات، زمان ته‌نشینی، قدرت میدان مغناطیسی خارجی و شرایط محیط اسیدی با pH 5 تا 6 از عوامل افزایش‌دهنده راندمان حذف کدورت توسط نانو ذرات مغناطیسی پوشش داده شده با کیتوزان بود. با کمک نانوذرات مورد مطالعه کدورت آب تا حد 5/99 درصد در شرایط بهینه کاهش یافت. بحث و نتیجه‌گیری: نانو ذرات مغناطیسی پوشش داده شده با کیتوزان، کارایی خوبی در حذف کدورت از محلول‌های آبی نشان داد. استفاده از یک میدان مغناطیسی خارجی توانست به طور موثر زمان ته‌نشینی فلوک‌های مغناطیسی را کاهش دهد. لذا کاربرد توام نانو‌ذرات مغناطیسی مورد مطالعه همراه با میدان مغناطیسی خارجی می‌تواند کدورت آب‌های بسیار کدر را به میزان قابل توجهی کاهش دهد.

Title: بررسی عملکرد فناوری اولتراسونیک و پراکسید هیدروژن در حذف بیس‌فنولA از محلول‌های آبی

http://tbj.ssu.ac.ir/article-1-2108-fa.html

مقدمه: بیس‌فنولA یک آنتی‌اکسیدان غیرقابل تجزیه بیولوژیکی بوده که مخاطرات زیادی برای سلامتی انسان و حیوانات دارد. و به علت اینکه تنها مقدار کمی از آن در طی تصفیه فاضلاب حذف می‌شود، فاضلاب حاوی بیس‌فنولA می‌تواند منبع آلودگی در محیط آبزی باشد. لذا هدف از انجام این مطالعه بررسی عملکرد فناوری اولتراسونیک و پراکسید هیدروژن در حذف بیس‌فنولA از محلول‌های آبی می‌باشد. روش بررسی: آزمایشات سونوشیمیایی توسط دستگاه اولتراسونیک )ساخت شرکت (Elma در توان‌‌های 300 و 500 وات و فرکانس‌‌های 35 و 130 کیلوهرتز در انجام شد. غلظت پراکسید هیدروژن 5، 15و 30 میلی‌گرم در لیتر و غلظت اولیه بیس‌فنولA در محدوده 2، 5، 20و 50 میلی‌گرم در لیتر بود. جهت اندازه‌گیری غلظت بیس‌فنولA از دستگاه Spectrometer UV/VIS Lambada 25 Perkin Elmer, Shelton استفاده‌شده است. یافته‌ها: نتایج نشان داد که فرآیند تلفیقی اولتراسونیک و پراکسید هیدروژن با راندمان 65/98 درصد، بالاترین راندمان را در حذف بیس‌فنولA دارا می‌باشد. بیشترین میزان حذف نیز در فرکانس 130 کیلوهرتز و توان 500 وات همراه با 30 میلی‌گرم‌ در لیتر پراکسید هیدروژن در 11pH= مشاهده شد. همچنین نتایج این مطالعه نشان داد که با افزایش pH سرعت تجزیه بیس‌فنولA توسط هر سه فرآیند (اولتراسونیک، پراکسیدهیدروژن و تلفیق اولتراسونیک و پراکسیدهیدروژن) افزایش می‌یابد. نتیجه‌گیری: نتایج این تحقیق نشان داد که فرآیند تلفیقی اولتراسونیک و پراکسید هیدروژن می‌تواند به‌عنوان یک روش پاک و دوستدار محیط‌زیست جهت تصفیه آب­‌های حاوی بیس‌فنولA در حد معقول مورداستفاده قرار بگیرد.

Title: بررسی نقش جلبک‌ها در بوی آب جشمه ساسان کازرون

http://tbj.ssu.ac.ir/article-1-2110-fa.html

مقدمه: ازآنجاکه بوی آب آشامیدنی ازنظر مصرف‌کنندگان ناخوشایند است، بنابراین در مناطقی که بوی آب مورد اعتراض مصرف‌کنندگان می‌باشد، بایستی تحقیقاتی جهت یافتن عامل ایجاد بو در منابع تأمین آن انجام گیرد تا بتوان راهکار‌های مهندسی و مدیریتی در جهت کاهش آن پیدا نمود. مشکل بو در چشمه ساسان کازرون نیز که از منابع تأمین آب آشامیدنی شهرهای کازرون، بوشهر و تعدادی از روستاهای شهرهای مربوطه می‌باشد. در چندین سال اخیر باعث اعتراض مصرف‌کنندگان شده است. با توجه به اینکه مطالعات، فلور جلبکی منابع آبی را یکی از عوامل مهم ایجاد بو نشان داده‌اند، در این مطالعه  نقش جلبک‌های مولد بو در بوی نامطبوع این چشمه موردبررسی قرار گرفت. روش بررسی: نمونه‌برداری‌های منظمی از آب چشمه صورت گرفت و پس از تعیین ((TON: Threshold Odor Number نوع جلبک‌ها تشخیص داده شد و سپس تعداد آن‌ها به روش شمارش مستقیم میکروسکوپی شمرده شد .با توجه به اینکه جرم توده جلبکی ارتباط مستقیم با غلظت نیتروژن و فسفر دارد، غلطت این عناصر که در سال‌های اخیر توسط نهادهای مسئول مورد آزمایش قرارگرفته بود بررسی گردید و با غلطت لازم این عناصر برای رشد جلبک‌ها مقایسه گردید. یافته‌ها: نتیجه مطالعات (TON) را برابر477/4 تا2/6  نشان داد که نشان از حد قابل‌تشخیص بو بود. نمودار ورگرسیون انجام‌شده ارتباط مستقیمی بین میزان  TONو تعداد جلبک‌ها نشان داد. مقدار غلظت نیترات و فسفات در آب چشمه شرایط مساعد رشد جلبک‌ها را تأیید می‌نمود. نتایج شناسایی جلبک‌ها نشان داد در آب چشمه جلبک اسیلاتوریا و میکروسیستیس از گروه سیانوباکتریها(جلبک‌های سبز-آبی) که از عوامل تولید‌کننده طعم و بو می‌باشد به تعداد زیاد وجود دارد که باعث ایجاد بوی خاکی- کپکی در آب می‌شود. بررسی نقشه‌های زمین‌شناسی نیز نشان داد که چشمه آبرفتی و متأثر از دشت طراف می‌باشد. نتیجه‌گیری: چون عامل بوی چشمه جلبک‌ها تشخیص داده شد و رشد جلبک‌ها وابستگی مستقیمی به وجود عوامل رشد دارد، و همچنین چشمه متأثر از نفوذ آلودگی‌های ناشی از کودهای بکار رفته در باغات اطراف است، لذا برای کنترل جلبک‌ها و بوی حاصله نیاز به مدیریت و کنترل استفاده از کودها و سایر آلاینده‌های حاوی ازت و فسفر می‌باشد.

Title: وضعیت اپیدمیولوژیک بیماری مالاریا در استان قم طی سال‌های 91-1380

http://tbj.ssu.ac.ir/article-1-2109-fa.html

مقدمه: مالاریا یکی از مهمترین مشکلات بهداشتی در بسیاری از مناطق جهان می‌باشد. این بیماری در 106 کشور جهان به صورت آندمیک وجود دارد. این مطالعه باهدف مشخص نمودن سیمای اپیدمیولوژیک مالاریا در استان قم طی سال‌های 91-1380 انجام شد. روش بررسی: این مطالعه به روش توصیفی- مقطعی بوده و با استفاده از داده‌های موجود بیماران مبتلابه مالاریا، طی سال‌های 91-1380 در استان قم انجام شد. داده‌ها با نرم‌افزار SPSS و آزمون‌های T و کای دو مورد تجزیه‌وتحلیل قرار گرفت. یافته‌ها: 599 مورد بیمار مبتلابه مالاریا از استان قم گزارش شدند. مردان با  3/64 درصد اکثریت مبتلایان را تشکیل داده بودند. بیشترین موارد آلودگی(31/91%) ناشی از پلاسمودیوم ویواکس بوده است. 5/8 درصد از مبتلایان به مالاریا، ایرانی و 5/91 درصد بقیه غیر ایرانی بودند. همچنین بیشترین موارد بیماری (11/67%) در گروه سنی 15 سال و بالاتر رخ‌داده است. نتیجه‌گیری: با توجه به زائرپذیر بودن استان قم و نیز با عنایت به وجود پشه‌های ناقل، برنامه‌های مبارزه با مالاریا نظیر بیماریابی و درمان بیماران می‌بایست تداوم داشته باشد

Title: اثرضد باکتریایی عصاره هسته انگور بر ماندگاری ماهی تیلاپیا

http://tbj.ssu.ac.ir/article-1-2112-fa.html

چکیده مقدمه: گیاهان دارویی به سبب دارا بودن ترکیبات آنتی‌باکتریایی طبیعی بسیار موردتوجه هستند و ایران نیز یکی از غنی‌ترین منابع گیاهان دارویی به شمارمی‌رود. هدف از این پژوهش، بررسی تأثیر آنتی‌باکتریایی عصاره هسته انگور برافزایش عمر ماندگاری ماهی تیلاپیا است. روش بررسی : ماهی‌ها بلافاصله پس از صید و عمل شستشو به دو بخش تقسیم شدند. یک گروه در عصاره هسته انگور (غلظت5/1%)به مدت 30 دقیقه غوطه‌ور و سپس در کیسه‌های پلی‌اتیلن بسته‌بندی گردیدند، دومین گروه نمونه‌ها نیز به‌عنوان شاهد بسته‌بندی و همه در دمای (1±4) درجه سانتی‌گراد یخچال نگهداری شدند. آزمایش‌های میکروبی شامل، شمارش کلی میکروب‌های هوازی مزوفیل (Totalcount)، باکتری‌های سرما‌گرا (PTC)، به همراه ارزیابی حسی در یک دوره 20 روزه در فواصل زمانی(2،0 ،5 ،10، 20،15)انجام شد. یافته‌ها: بر اساس نتایج حاصل، گروه حاوی عصاره به‌طور معنی‌داری (05/0P<) فساد میکروبی را نسبت به نمونه شاهد به تعویق انداخت، بطوریکه تعداد باکتری‌های شمارش‌شده در ماهی‌های تیمار شده با عصاره هسته انگور تا پایان دوره نگهداری کمتر از حد قابل‌قبول پیشنهادی (log cfu/g 7)باقی ماند.همچنین در بررسی حسی ماهیان تیمار‌شده با عصاره هسته انگور بالاترین کیفیت را در طول دوره نگهداری از خود نشان دادند. نتیجه‌گیری: ماهی تیمارشده با عصاره هسته انگور در این مطالعه تا انتهای دوره نگهداری قابل‌مصرف بود، به‌طوری‌که عصاره توانست عمر ماندگاری نمونه‌ها را نسبت به نمونه شاهد  افزایش دهند.

Title: بررسی میزان آگاهی بیماران مراجعه‌کننده به مراکز تصویربرداری بیمارستان‌های استان یزد در مورد خطرات پرتوهای یونیزان در سال 1393

http://tbj.ssu.ac.ir/article-1-2111-fa.html

مقدمه: بیماران باید از خطرات پرتوهای یونیزان مورداستفاده در آزمون‌های تصویربرداری پزشکی مطلع باشند تا بتوانند تصمیم‌های مناسبی درروند تشخیص و درمان بیماری خود داشته باشند. هدف از انجام این مطالعه بررسی میزان آگاهی بیماران مراجعه‌کننده به مراکز تصویربرداری بیمارستان‌های استان یزد در مورد خطرات انجام آزمون‌های رادیولوژی و سی‌تی‌اسکن می‌باشد. روش بررسی: این مطالعه از نوع توصیفی- مقطعی است که بر روی 251 نفر بیمار از فروردین تا خرداد 1393 انجام شد. پرسشنامه‌ای با 33 سؤال شامل سؤالاتی در زمینه اطلاعات جمعیت‌شناسی، آگاهی از روش‌های مختلف تصویربرداری پزشکی و خطرات احتمالی مرتبط با آن و همچنین سؤالاتی درباره انتظارات بیماران از پزشکان در بین بیماران توزیع شد و از آن‌ها خواسته شد که آن را تکمیل نمایند.  یافته‌ها: میانگین نمره مربوط به آگاهی بیماران 21/56 درصد و انحراف معیار برابر با 78/16 درصد بود. رابطه معنی‌داری بین سن، جنسیت و مدرک تحصیلی بیماران با میزان آگاهی آن‌ها مشاهده شد. درحالی‌که 6/95 درصد بیماران انتظار داشتند که پزشکشان در مورد منافع و خطرات احتمالی هر آزمون به آن‌ها توضیح دهد ولی فقط 6 درصد پزشکان در این مورد به آن‌ها توضیح داده بودند. نتیجه‌گیری: آگاهی بیماران می‌تواند نقش مهمی در کاهش انجام آزمون‌های تصویربرداری غیر‌ضروری ایفا نمایند ولی باوجود تمایل فراوانی که در بین بیماران جهت کسب آگاهی در این زمینه وجود دارد، میزان آگاهی آن‌ها در این موضوع مطلوب نمی‌باشد. بنابراین باید با اتخاذ راهکارهایی آگاهی بیماران را افزایش داد.

Title: بررسی تعیین‌کننده‌های رفتار پیشگیری‌کننده از عفونت ادراری در زنان باردار براساس سازه‌های مدل اعتقاد بهداشتی در شهر یزد

http://tbj.ssu.ac.ir/article-1-2113-fa.html

مقدمه: عفونت ادراری یکی از شایع‌ترین عوارض بارداری به حساب می‌آید، که با به کارگیری رفتارهای پیشگیری‌کننده می‌توان از وقوع آن جلوگیری کرد. هدف از مطالعه حاضر تعیین رفتارهای پیشگیری‌کننده از عفونت ادراری در زنان باردار براساس سازه‌های مدل اعتقاد بهداشتی در شهر یزد بود. روش‌بررسی: این مطالعه توصیفی- مقطعی، بر روی 160 نفر از زنان باردار مراجعه کننده به مراکز بهداشتی درمانی شهر یزد انجام شد، که مبتلا به عفونت ادراری بودند. ابزار گرد‌آوری داده‌ها پرسشنامه مبتنی بر ساختارهای مدل اعتقاد بهداشتی بود. داده‌های حاصل با استفاده از نرم‌افزار SPSS نسخه 18 و جداول توزیع فراوانی بیان وبا آزمون‌های تی تست تجزیه و تحلیل شد. یافته‌ها: در میان زنان باردار مورد مطالعه 1/63% (101 نفر)  نمره آگاهی متوسط و 9/36% (59 نفر)  نمره آگاهی خوب داشتند. از نظر نمره سازه‌های مدل اعتقاد بهداشتی1/73% ( 117 نفر)  نمره موانع درک شده خوب، 95% (152 نفر)  نمره منافع درک شده خوب، 95% (152 نفر) نمره حساسیت درک شده خوب و 3/81% (130 نفر ) نمره شدت درک شده خوب داشتند. بین نمره کلی آگاهی با نگرش همبستگی معنی‌داری وجود داشت(02/0p=) ولی همبستگی آگاهی با رفتار(08/0p=) و همبستگی نگرش با رفتار(09/0p=) معنی‌دار نبود. نتیجه‌گیری: با توجه به نتایج نیاز است که در مداخلات آتی بر سازه موانع درک‌شده در جهت پیشگیری از عفونت ادراری تمرکز شود و همچنین رفتارهای مربوط به عادات غذایی و نظافت بیشتر مد نظر قرار گیرد، همچنین لزوم مشارکت خانواده، همسر و رسانه‌های جمعی در طراحی برنامه‌های آموزشی ضروری است.

Title: بررسی وضعیت شاخص DMFT در بیماران دیابتی نوع دو و رابطه آن با HbA1C در شهر یزد در سال 1392

http://tbj.ssu.ac.ir/article-1-2114-fa.html

مقدمه: دیابت منجر به عوارض و مشکلات متعددی می‌ شود که مشکلات دهانی یکی از مهم‌ترین مشکلات بهداشتی در این بیماران می‌باشند. مطالعه حاضر با هدف تعیین وضعیت شاخص DMFT در بیماران دیابتی نوع دو و رابطه آن  با HbA1c در شهر یزد در سال 1392 انجام شد. روش بررسی: در این مطالعه توصیفی- مقطعی، 203 نفر از بیماران دیابتی نوع دو که در سال 1392 به مرکز تحقیقات دیابت یزد مراجعه کرده بودند، در دو گروه دیابت کنترل شده و نشده (HbA1c کمتر یا بیشتر از 7%) به صورت تصادفی ساده انتخاب و مورد مطالعه قرار گرفتند.اطلاعات مورد نیاز با استفاده از پرسشنامه ای که شامل شاخص DMFT و HbA1C به انضمام متغیرهای دموگرافی بود، جمع آوری گردید. داده های با استفاده از نرم افزار آماری 17SPSS آزمون های Chi-square، Fisher exact test، ANOVA ، T-test و ضریب همبستگی مورد تجزیه و تحلیل قرار گرفتند. یافته ها:یافته های این پژوهش نشان داد میانگین تعداد دندان های پوسیده و از دست رفته و ترمیم شده به طور معنی داری در گروه بیماران با دیابت کنترل نشده بالاتر می باشد(000/0=P). بین شاخص DMFT در دو گروه مورد بررسی بر حسب استفاده از مسواک و نخ دندان نیز اختلاف آماری معناداری وجود داشت (000/0=P) نتیجه گیری:  شاخص DMFT در بیماران با دیابت کنترل نشده به طور معناداری بیشتر از گروه بیماران با دیابت کنترل شده بود. بنابر این لازم است به بیماران دیابتی کنترل نشده در این زمینه آگاهی داده شود.

Title: بررسی تأثیر آموزش رفتارهای مدیریت تغذیه از طریق پیام‌کوتاه مبتنی‌بر الگوی اعتقاد بهداشتی

http://tbj.ssu.ac.ir/article-1-2115-fa.html

مقدمه: پیام متنی می‌تواند به عنوان راهی برای ارائه برنامه‌های آموزشی پیشگیری مبتنی برالگوهای تغییر رفتار نظیر تئوری رفتار برنامه‌ریزی شده و مدل اعتقاد بهداشتی استفاده شود و می‌تواند به عنوان رویکرد جایگزینی نسبت به برنامه‌های آموزش فردی وگروهی در نظرگرفته شود. مطالعه حاضر به منظور بررسی اثربخشی این روش نوین در ارتقاء رفتارهای مدیریت غذایی برگروهی از رابطین بهداشتی شهر یزد مبتنی بر الگوی باور سلامتی صورت گرفت. روش بررسی: در این مطالعه مداخله ای تعداد 200 نفر از رابطین بهداشتی که در 16 مرکز بهداشتی درمانی شهر یزد مشغول فعالیت بودند شرکت داشته که بطور تصادفی و یکسان به دو گروه مداخله و کنترل تقسیم شدند. پس از برگزاری پیش آزمون برای هر دو گروه، مداخله آموزشی بوسیله یک بسته آموزشی با تعداد تقریبی 500 پیامک در زمینه رفتارهای مدیریت تغذیه مبتنی بر الگوی باور سلامتی در مدت یک ماه و نیم به صورت روزانه ارسال شد و بعد از گذشت 8 هفته از مداخله پس از آزمون بعمل آمد. ابزار جمع‌آوری اطلاعات، پرسش نامه‌ای مبتنی بر سازه‌های الگوی باور سلامتی به انضمام متغیرهای جمعیت‌شناختی بود. داده‌ها با استفاده از نرم افزار SPSS نسخه 18 و با بکارگیری آمارتوصیفی، آزمون‌های آماری Non Parametric Wilcoxon، Mann-Whitney و Kruskal-Wallis، ²X و RR مورد تجزیه و تحلیل قرار گرفت. یافته‌ها: رفتار کلی مدیریت غذا، بعد از آموزش در گروه مداخله به طور معنی‌داری افزایش یافت، درحالی که در گروه کنترل افزایش معنی‌داری مشاهده نشد. رفتار پختن، بعد از آموزش در گروه مداخله و همچنین گروه کنترل افزایش معنی‌داری نداشت. رفتار بهداشت فردی، بعد از آموزش در گروه مداخله و همچنین گروه کنترل افزایش معنی‌داری نداشت. رفتار جداسازی، بعد از آموزش در گروه مداخله افزایش معنی‌داری نداشت، درحالی که در گروه کنترل به طور معنی‌داری افزایش یافت. میانگین نمره سازه‌های حساسیت، شدت، منافع و موانع درک‌شده بعد از آموزش در گروه مداخله به طور معنی‌داری افزایش یافت، درحالی که در گروه کنترل به جز سازه شدت درک‌شده که بطور معنی‌داری کاهش یافته بود. نتیجه‌گیری: سرویس پیام کوتاه می تواند به عنوان یک روش جدید و مؤثر در شکل‌دهی به رفتارهای مدیریت غذایی و بویژه تعیین‌کننده‌های آن مبتنی‌بر الگوی باور سلامتی استفاده گردد.

Title: اثر بخشی شناخت درمانی گروهی برکیفیت زندگی و شادکامی مادران کودکان مبتلا به اختلال اوتیسم

http://tbj.ssu.ac.ir/article-1-2117-fa.html

چکیده مقدمه: بررسی ها نشان می دهند که والدین کودکان با اختلالهای گسترده اوتیسم بیش از والدین کودکان بااختلالهای روان شناختی دیگر، در معرض فشارهای روانی ناشی از داشتن کودک ناتوان قرار دارند.تمامی مسائل و مشکلات این کودکان می تواند کیفیت زندگی و شادکامی خانواده های این کودکان را به خطر اندازد. لذا پژوهش حاضر با هدف اثربخشی روش شناخت درمانی گروهی بر کیفیت زندگی و شادکامی مادران کودکان مبتلا به اوتیسم در شهر شیراز صورت گرفت. روش بررسی: پژوهش حاضر ازنوع شبه آزمایشی به صورت پیش آزمون-پس آزمون همراه با گروه کنترل می باشد. بدین منظور ابتدا بر روی جامعه (130 نفری والدین اوتیسم در شهر شیراز)پرسشنامه های Happiness و quality of life اجرا و پس از بررسی نتایج به صورت تصادفی تعداد 30 نفر از مادرانی که نمره کمتری درپرسشنامه ها کسب کرده بودند انتخاب و در دو گروه آزمایش(15 نفر) و گواه(15 نفر) قرار گرفتند.روش شناخت درمانی گروهی بر روی گروه آزمایش به مدت 5/2 ماه طی 12 جلسه 5/1 ساعته اجرا گردید و طی همین مدت بر روی گروه گواه روش خاصی اجرا نشد،  سپس پس آزمون اجرا گردید، آزمون پیگیری بعد از دو ماه اجرا گردید. داده ها از طریق آمار توصیفی(درصد،  فراوانی،  میانگین، انحراف معیار)و استنباطی (آزمون تی و کواریانس) مورد بررسی و تعبیر و تفسیر قرارگرفت. یافته ها: نتایج نشان داد که شناخت درمانی گروهی در گروه آزمایش به طور معناداری باعث افزایش کیفیت زندگی و ابعادآن  و میزان شادکامی مادران شده است (001/p< ) همچنین شناخت درمانی گروهی در مرحله پیگیری تاثیر معناداری بر کیفیت زندگی و شادکامی داشته است(001/p<) نتیجه گیری: نتایج این پژوهش نشان دادکه مداخله موجب بهبود کیفیت زندگی و ابعاد آن در والدین کودکان مبتلا به اختلال اوتیسم می گردد که در نتیجه آن میزان شادکامی والدین افزایش یافته است.

Title: بازیافت لجن مازاد تصفیه‌خانه فاضلاب با کاربرد آن به عنوان کربن فعال پودری جهت حذف متیل ترشیری بوتیل اتر (MTBE) از محیط‌های آبی

http://tbj.ssu.ac.ir/article-1-2116-fa.html

مقدمه: امروزه استفاده رو به گسترش از MTBE و همچنین خواص فیزیکی و شیمیایی آن از جمله حلالیت بسیار بالا در آب، باعث ورود این ماده به منابع آب می‌شود. از طرف دیگر لجن فاضلاب شهری حاوی موادی است که عمده آن‌ها حاوی درصد کربن بالایی نیز می‌باشند. لذا هدف از این مطالعه تهیه کربن فعال از لجن تصفیه‌خانه فاضلاب شهری و بررسی کارایی آن جهت حذف MTBE می‌باشد. روش بررسی: پس از تهیه زغال از لجن تصفیه‌خانه و فعال کردن آن به وسیله محلول 3 مولار از H2PO4 وKOH  و محلول 5 مولار از ZnCl2، اثر پارامترهایی چون نوع فعال‌کننده، pH (10-2)، زمان تماس (min240-0)، دوز جاذب (g/L6-2) و غلظت اولیه MTBE (mg/L70-20) در جذب MTBE بر کربن ساخته شده تعیین گردید. غلظت باقی مانده MTBE در محلول با استفاده از دستگاه گازکروماتوگرافی تعیین شد. در پایان داده‌های تجربی بدست آمده با استفاده از مدل‌های جذبی فروندلیخ و لانگموئیر مدلسازی شدند. یافته ها: هرچند از لحاظ آماری تفاوت معنی داری در استفاده از فعال کننده‌های مختلف حاصل نگردید، اما بیشترین جذب MTBE در هر گرم از کربن فعال هنگامی حاصل می‌شود که از ZnCl2 به عنوان فعال کننده استفاده شد. بیشترین راندمان حذف (حدود %50) در pH اسیدی 4، دوز g/L6 از کربن فعال و غلظت mg/L20 از MTBE حاصل شد. نتایج ایزوترم‌های جذب نشان داد که مدل جذبی فروندلیخ تطابق بیشتری با داده‌های تجربی دارد. نتیجه گیری: با توجه به مشکلاتی که در دفع لجن تصفیه‌خانه‌های فاضلاب وجود دارد، بازیافت آن به عنوان یک جاذب می‌تواند بسیاری از این مشکلات را مرتفع سازد. لذا پیشنهاد می‌شود کارایی این ماده در حذف آلاینده‌های دیگر و بررسی‌های اقتصادی جهت کاربرد آن در مقیاس صنعتی مورد بررسی قرار گیرد.

Title: مقایسه مدل حاصل از رگرسیون و مدل کینتیکی اصلاح شده مونود در پیش بینی راندمان حذف فرمالدوئید در بیوفیلتر چکنده

http://tbj.ssu.ac.ir/article-1-2118-fa.html

چکیده مقدمه: فرمالدوئید یکی از ترکیبات آلاینده سمی، جهش زا و احتمالاً سرطانزا می‌باشد که از صنایع مختلف دنیا به میزان زیاد به هوا تخلیه می‌گردد. هدف اصلی از این مطالعه بررسی پارامترهای کینتیکی یک بیوفیلتر چکنده و در کنار آن ارائه مدلی ساده رگرسیونی می‌باشد. روش بررسی: در این مطالعه با کمک داده‌های حاصل از یک بیوفیلتر صافی چکنده در مقیاس آزمایشگاهی که در حذف بخار فرمالدوئید از هوا مورد استفاده قرار گرفته بود جهت تعیین ضرایب کینتیکی Km و rmax اقدام گردید. همچنین به کمک این داده‌ها یک مدل رگرسیونی ساده نیز محاسبه شد. یافته‌ها: در نهایت با کمک این معادلات نسبت به پیش بینی راندمان سیستم در غلظت‌های مختلف ورودی به بیوفیلتر اقدام گردید و داده‌های حاصل با اندازه‌گیری‌های واقعی در پایلوت مورد مقایسه قرار گرفت.  نتیجه‌گیری: نتایج حاصل از این مطالعه مشخص نمود که مدل رگرسیونی از دقت بالایی بر خوردار است لیکن فقط قادر به پیش بینی متوسط راندمان بیوفیلتر در حذف بخار فرمالدوئید بود. ولیکن مدل کینتیکی دارای مقداری خطا در پیش‌بینی‌ها بود لیکن نتایج این مدل اتطباق زیادی با نتایج ورودی داشته و تقریباً فراز و فرودهای دوره راهبری سیستم را پیش‌بینی نمود.

Title: طراحی ساختار اجرایی اعتباربخشی بیمارستانها از دیدگاه ذی‌نفعان

http://tbj.ssu.ac.ir/article-1-2119-fa.html

مقدمه :موفقیت هر سازمانی در گرو وجود یک نظام ارزشیابی کارآمد است. استفاده از استانداردهای اعتباربخشی بسیار کمک‌کننده است. اعتباربخشی به معنی فرآیند ارزیابی خارجی جهت بررسی سطح عملکرد مراکز بهداشتی درمانی می‌باشد. پژوهش حاضر با هدف طراحی ساختار اجرایی اعتباربخشی از دیدگاه ذی‌نفعان انجام پذیرفت. روش بررسی: مطالعه توصیفی کاربردی حاضر به صورت مقطعی در سال 93-92 با مشارکت 200 نفر از ذی‌نفعان اعتباربخشی بیمارستان سراسر کشور انجام شده است. منظور از ذی‌نفعان کسانی هستند که درگیر اجرای اعتباربخشی هستند و از ساختار فعلی و هدف آن آگاهی دارند. ابزار بررسی در این پژوهش پرسشنامه بسته است. در فاز اول پرسشنامه نیمه باز توسط 72 نفر تکمیل گردید که پس از اضافه نمودن نظراتشان، پرسشنامه به صورت بسته دراختیار 128 نفر دیگر از ذی‌نفعان قرار گرفت. افراد به سوالات که در رابطه با ابعاد اجرای اعتباربخشی بود، در طیف10-0 امتیاز دادند. روایی داده ها توسط صاحبنظران و پایایی آن ازطریق آزمون آلفای کرونباخ 2/96 به دست آمد و با نرم افزار SPSS نسخه 18 مورد تجزیه تحلیل قرار گرفت. یافته‌ها:یافته‌ها نشان داد اکثر ذی‌نفعان در رابطه با ساختار کلی اجرایی اعتباربخشی معتقدند بیمارستانها باید در ابتدا به صورت داوطلبانه و سپس اجباری توسط یک ارگان مستقل غیر دولتی مانند JCI با استانداردهای بین‌المللی بومی شده، به صورت سالانه و به همراه آموزش پیش از اعتباربخشی مورد ارزیابی قرارگیرند. نتیجه گیری:بطور کلی ذی‌نفعان با شیوه اعتباربخشی کنونی موافق نبوده و براساس نتایج بررسی توصیفی و تحلیلی نظرسنجی پاسخگویان مورد پژوهش، ساختار نهایی اجرایی اعتباربخشی ارائه گردیده است.

Title: اثربخشی روان‌درمانگری اسلامی مبتنی بر کیفیت‌زندگی بر افسردگی و کیفیت‌زندگی دانشجویان (دختر و پسر) افسرده دانشگاه پیام نور استان چهارمحال و بختیاری

http://tbj.ssu.ac.ir/article-1-2120-fa.html

چکیده مقدمه: یکی از اولویت‌های نظام سلامت توجه به بهداشت روان است. از طرفی در سالیان اخیر، پرداختن به مذهب و معنویت به عنوان یکی از مؤلفه‌های اساسی رویکردهای درمانی و آموزشی، مورد تأکید قرار گرفته‌است. این پژوهش با هدف بررسی اثربخشی روان‌درمانگری مبتنی بر کیفیت‌زندگی با جهت گیری اسلامی، بر افسردگی و کیفیت زندگی دانشجویان دانشگاه پیام نور استان چهارمحال و بختیاری انجام شده است. روش بررسی: پژوهش حاضر، در قالب یک طرح نیمه‌آزمایشی انجام شده است. برای این کار، پس از انجام مصاحبه تشخیصی 40 نفر از دانشجویان افسرده مراجعه کننده به هسته‌های مشاوره دانشگاه در سال تحصیلی 93-94 انتخاب و بعد از اخذ رضایت آنان جهت شرکت در پژوهش، به روش جایگزینی تصادفی در دو گروه آزمایش و کنترل گمارده شدند. جهت سنجش متغیرها، از پرسشنامه‌های افسردگی بک و پرسشنامه کیفیت زندگی سازمان بهداشت جهانی استفاده شد. هر دو گروه در پیش‌آزمون شرکت نمودند و سپس گروه آزمایش در 10 جلسه و هر جلسه به طور متوسط 90 دقیقه به صورت گروهی، مداخله‌ درمانی را دریافت نمودند. برای تحلیل یافته‌ها از روش تحلیل کواریانس و نرم افزار SPSS-18 استفاده شده است. یافته‌ها: نتایج نشان داد روان‌درمانگری اسلامی مبتنی بر کیفیت‌زندگی کاهش معنادار افسردگی(73/0= 2η ،001/0>p، 3/96= (1،33) f) و افزایش کیفیت‌زندگی(52/0= 2η ،001/0>P، 77/35= (1،34) f)  را در گروه آزمایش به همراه داشته است. نتیجه‌گیری: پرداختن به بعد مذهب به عنوان یکی از مهمترین مؤلفه‌های تشکیل دهنده‌ فرهنگ، و افزودن این بعد به مداخلات روانشناختی، با نگاهی کل‌گرایانه و یکپارچه نسبت به انسان می‌تواند به اثربخشی مداخلات کمک کند.
